# Enhanced Performance of Cyclopentadithiophene-Based Donor-Acceptor-Type Semiconducting Copolymer Transistors Obtained by a Wire Bar-Coating Method

**DOI:** 10.3390/polym14010002

**Published:** 2021-12-21

**Authors:** Doyeon Kim, Minho Yoon, Jiyoul Lee

**Affiliations:** 1 Department of Smart Green Technology Engineering, Pukyong National University, Busan 48513, Korea; dy6425@naver.com (D.K.); minhoyoon78@gmail.com (M.Y.); 2 Department of Nanotechnology Engineering, Pukyong National University, Busan 48513, Korea

**Keywords:** cyclopentadithiophene-based conjugated polymer, polymer field-effect transistors, wire-bar coating, charge transport, Gaussian density of states (DOS)

## Abstract

Herein, we report the fabrications of high-performance polymer field-effect transistors (PFETs) with wire bar-coated semiconducting polymer film as an active layer. For an active semiconducting material of the PFETs, we employed cyclopentadithiophene-*alt*-benzothiadiazole (CDT-BTZ) that is a D-A-type-conjugated copolymer consisting of a repeated electron-donating unit and an electron-accepting unit, and the other two CDT-based D-A-type copolymer analogues are cyclopentadithiophene-*alt*-fluorinated-benzothiadiazole (CDT-FBTZ) and cyclopentadithiophene-*alt*-thiadiazolopyridine (CDT-PTZ). The linear field-effect mobility values obtained from the transfer curve of the PFETs fabricated with the spin-coating were 0.04 cm^2^/Vs, 0.16 cm^2^/Vs, and 0.31 cm^2^/Vs, for CDT-BTZ, CDT-FBTZ, and CDT-PTZ, respectively, while the mobility values measured from the PFETs with the wire bar-coated CDT-BTZ film, CDT-FBTZ film, and CDT-PTZ film were 0.16 cm^2^/Vs, 0.28 cm^2^/Vs, and 0.95 cm^2^/Vs, respectively, which are about 2 to 4 times higher values than those of the PFETs with spin-coated films. These results revealed that the aligned molecular chain is beneficial for the D-A-type semiconducting copolymer even though the charge transport in the D-A-type semiconducting copolymer is known to be less critical to the degree of disorder in film.

## 1. Introduction

In recent decades, solution-processable polymer field-effect transistors (PFETs) based on conjugated copolymers with donor-acceptor (D-A)-type moieties, such as indacenodithiophene (IDT) [1,2], diketopyrrolopyrrole (DPP) [3,4], isoindigo (IDG) [5,6], naphthalenediimide (NDI) [7,8,9], and cyclopentadithiophene (CDT) [10,11,12], have received extensive attention due to their remarkably high field-effect mobilities exceeding 10 cm^2^ V^−1^ s^−1^ and low-energy bandgap characteristics. Rigid and nearly torsion-free planar polymer backbones in the D-A-conjugated copolymers have been regarded to be attributed to the enhanced charge transport along the chain and efficient interchain charge transfer through the strong π-π interaction between adjacent D-A subunits [13,14,15]. Several approaches, such as designing the D-A building blocks for controlling the push-pull strength of donor and acceptor moieties [16,17] and modification of the side chains [18], have been suggested for improving the molecular-structure of D-A-conjugated copolymers and electrical properties. However, polymers inherently have the twisted or tangled structures, of which disordered phases inevitably exist in the films [19,20,21,22]. Since the disordered phase impedes the charge transport due to the trap sites, it is necessary to minimize the twisting or tangle of the polymer molecular chains present in the polymer thin film channel of the PFETs and to align them well to obtain the PFETs with higher performance.

In this study, we report that the structural and electrical characteristics of the bar-coated polymeric films with cyclopentadithiophene-*alt*-benzothiadiazole (CDT-BTZ) and the other two CDT-based D-A-type copolymers of cyclopentadithiophene-*alt*-fluorinated-benzothiadiazole (CDT-FBTZ) and cyclopentadithiophene-*alt*-pyridine thiadiazole (CDT-PTZ). Bar coating is a widely used, large-area coating method for polymer films [23]. Through this bar coating, which allows the polymeric films to be gradually dried from the edge to center, aligned macroscopic structures and nano-fibril microstructures with large grains have been successfully formed [20]. Moreover, the bar-coated PFETs show the enhanced p-type charge-transport characteristics: The hole currents have been significantly increased with the threshold shifts and field-effect mobilities have been improved by factors of 2~4. The temperature-variable current-voltage analyses of the bar-coated PFETs reveals that the molecular ordering leads to the trap states of the polymeric films to be significantly minimized, and results in the high-performance PFETs. Therefore, it is strongly believed that the well-ordered polymer structures with enhanced electrical characteristics of the CDT-based films can be achieved by the bar-coating method.

## 2. Experimental Methods

**Figure 1:**
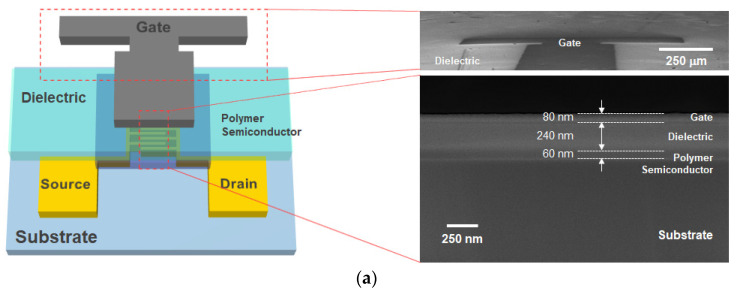

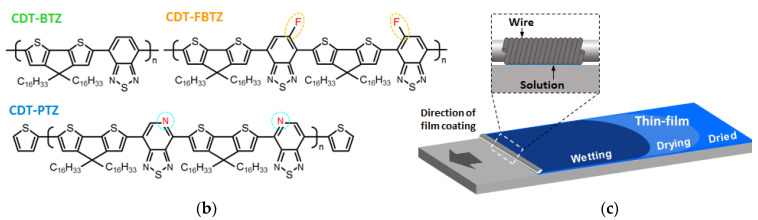
(**a**) Device configuration of the PFET used in this study and corresponding SEM images. (**b**) Molecular structures of the D-A-type semiconducting copolymers. (**c**) Schematic illustration of the wire-bar-coating process.

Top-gate, bottom-contact PFETs were fabricated, as shown in Figure 1a. A chemically cleaned soda-lime glass (Corning EAGLE Glass) was used as a substrate, and Ti (5 nm)/Au (35 nm) was thermally evaporated onto the substrate and patterned using conventional lift-off methods for source/drain electrodes. The channel width and length of PFETs were 1000 and 20 μm, respectively. The conjugated semiconducting copolymers (i.e., CDT-BTz (Mw (~60,000) with a polydispersity index (PDI) of 2.5), CDT-FBTz (Mw (~75,000) with a PDI of 2.5), and CDT-PTz (Mw (~60,000) with a PDI of 2.5)) were purchased from ONE-Materials Inc. (Dorval, QC, Canada) and used as received. The chemical structures of the CDT-based D-A-type copolymers are shown in Figure 1c. To fabricate the thin-film coatings, the semiconducting polymer solutions dissolved in chloroform at a concentration of 10 mg/mL were deposited using bar coating, as shown in Figure 1c, and subsequently thermally annealed at 200 °C for 60 min in nitrogen ambient, resulting in a 60-nm-thick semiconducting polymer film. Next, for the top-gate dielectric, PMMA (dissolved in n-butyl acetate at a concentration of 80 mg/mL) was spin-coated and cured at 80 °C for 60 min. The thickness of the PMMA was measured to be 240 nm, and its geometric capacitance was 6.86 nF cm^−2^ at 1 kHz. Finally, the top-gate electrode was formed by thermally evaporating an 80-nm-thick layer of Al through a shadow mask. The film thicknesses were confirmed using Field Emission Scanning Electron Microscope (FE-SEM) (MIRA 3, TESCAN).

The current-voltage (*I*-*V*) characteristics of the PFETs were characterized using a Keithley 236 Source Measure Unit in combination with a Keithley 2635 Source Meter controlled using a LabVIEW code. Capacitance-voltage (*C*-*V*) measurements of the polymer dielectric layers were recorded using an LCR meter (HP4284A, Agilent Technologies, Santa Clara, CA, USA). A liquid nitrogen cooling cryostat was used for the temperature-dependent (160–300 K) *I*-*V* measurements. The field-effect mobility of the FETs in the linear regime was found using the following equation:

(1) $$\mu_{lin}=\frac{1}{C_{i}V_{ds}}\frac{L}{W}\frac{\partial I_{ds}}{\partial V_{gs}},$$

where *I_ds_* is the drain current, *V_gs_* is the gate voltage, *C_i_* is the geometric dielectric capacitance, *V_ds_* is the drain voltage, and *L* and *W* are the channel length and width, respectively. All the electrical signals of the PFETs were measured in the dark.

## 3. Results and Discussion

**Figure 2:**
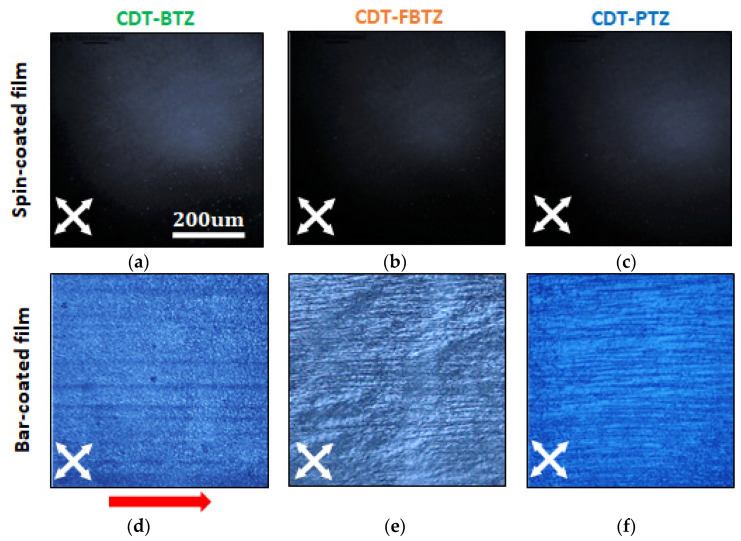
Cross-polarized optical microscope images of spin- and bar-coated films. (**a**,**d**) CDT-BTZ, (**b**,**e**) CDT-FBTZ, and (**c**,**f**) CDT-PTZ films. Red arrow is wire-bar coating direction.

**Figure 3:**
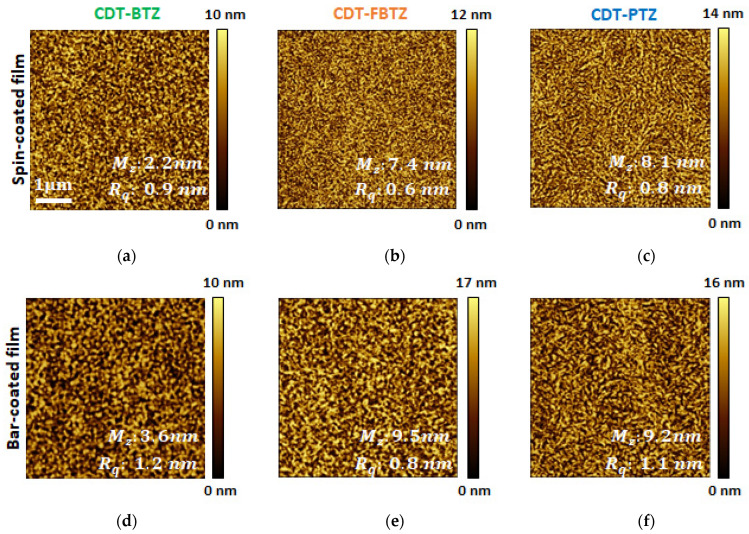
AFM images of spin- and bar-coated films of (**a**,**d**) CDT-BTZ, (**b**,**e**) CDT-FBTZ, and (**c**,**f**) CDT-PTZ films.

Figure 2a–f displays the cross-polarized optical microscope images of spin- and bar-films in Figure 2d–f, the aligned polymeric structure, in accordance with the coating direction, can be observed. Spin coating is a widely used, solution-processable, thin-film deposition method using centrifugal force, of which the thickness of the film can be determined by the spinning speed and viscosity of the solution. However, the instantaneous solvent evaporation during the process leads to the polymeric films to be hardly aligned. In contrast, as schematically illustrated in Figure 1b, the bar coating uses the wound-wire bar along a fixed substrate, which enables the wet film to be gradually dried from the edge to the center [20,21]. Thus, the polymeric films could be confined and uniaxially aligned, as observed. In addition, when the microscopic surface morphologies of the films were investigated with atomic force microscopy (AFM), in Figure 3, nano-fibril microstructures with large grains were definitely found in the bar-coated films. Although the directional structures, as in Figure 2, were hardly found, probably due to the polymeric molecular structure of the long chain and high molecular weight, it was clearly evident that the bar-coating method offers the larger, grainy, micro-film structures, which obviously attributed to the gradual drying from the edge to the center. The mean grain sizes for the bar-coated CDT-PTZ, CDT-FBTZ, and CDT-BTZ films were estimated to be 3.6, 9.5, and 9.2 nm, whereas those of the spin-coated films were 2.2, 7.4, and 8.1 nm, respectively. In addition, the root mean square (rms) surface roughness of the bar-coated films clearly increased and were measured to be 1.2, 0.8, and 1.1 nm for the bar-coated CDT-PTZ, CDT-FBTZ, and CDT-BTZ films, whereas it was 0.9, 0.6, and 0.8 nm for the spin-coated films, respectively.

**Figure 4:**
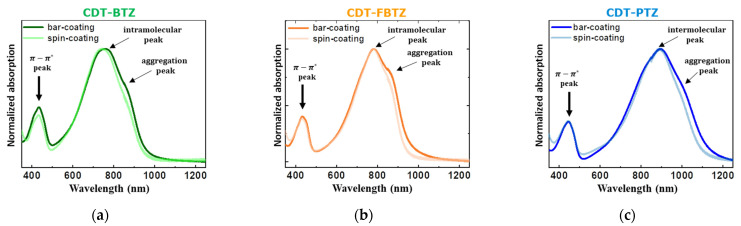
UV-vis absorbance spectra for spin- and bar-coated films of (**a**) CDT-BTZ, (**b**) CDT-FBTZ, and (**c**) CDT-PTZ films.

Moreover, UV/Vis absorption spectra of the films clearly showed the bar-coating-driven structural ordering in the CDT-based polymer films. As shown in Figure 4, all the D-A polymer films exhibited a high energy peak between 400 to 500 nm, corresponding to the π-π* transition, and a low energy peak between 700 to 1000 nm, which was attributed to the intramolecular charge transition [24,25,26]. More noteworthy, aggregation peaks, which were located at 830, 835, and 1000 nm, were identified for the bar-coated CDT-BTZ, CDT- FBTZ, and CDT-PTZ films. Furthermore, the intramolecular peaks were clearly red-shifted by 12, 6, and 6 nm for the films. As reported in [27], the emergence of those additional peaks and the red shifts was regarded to be attributed to the molecular aggregation. By stacking with the same adjacent polymer sub-units, the relevant energy states of the conjugated polymers (additional peaks) were formed, thereby resulting in decreases in the optical band gap of the polymeric films.

**Figure 5:**
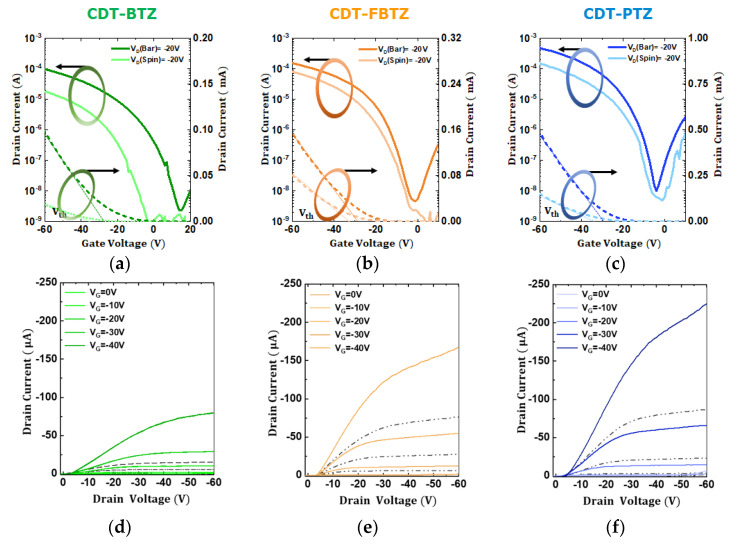
Linear regime transfer characteristics of the bar-coated and spin-coated (**a**) CDT-BTZ-, (**b**) CDT-FBTZ-, and (**c**) CDT-PTZ-based PFETs in the linear regime (*V_DS_* = −20 V). The output curves of the (**d**) CDT-BTZ-, (**e**) CDT-FBTZ-, and (**f**) CDT-PTZ-based PFETs with the bar-coating and spin-coating.

Next, top-gate, bottom-contact PFETs were fabricated and their current-voltage characteristics were investigated by measuring 20 samples at each condition (see Figure S1 of the Supplementary Materials). Figure 5a–c and Figure 5d–f, respectively, display the transfer characteristics (*I_ds_* vs. *V_gs_*) of PFETs with spin-coated and bar-coated CDT-BTZ, CDT, FBTZ, and CDT-PTZ films and the corresponding output characteristics (*I_ds_* vs. *V_ds_*) of the PFETs. In all the cases of the CDT-based-PFETs, hole conduction of the bar-coated PFETs was remarkably enhanced. Compared to that of the spin-coated PFETs, the hole currents (*I_ds_* at *V_gs_* = −60 V) of the bar-coated PFETs increased by an order of magnitude, and the threshold voltages (*V_th_*) of the PFETs were shifted to the depletion mode, which indicates the enhanced p-type conduction. The maximum transconductance of the bar-coated CDT-BTZ, CDT-FBTZ, and CDT-PTZ PFETs was estimated to be 3.1, 4.9, and 17 μs, respectively, whereas those of the spin-coated PFETs were deduced to be 0.6, 3.0, and 6.2 μs. Meanwhile, the linear field-effect hole mobility of the spin-coated CDT-BTZ, CDT-FBTZ, and CDT-PTZ PFETs was estimated to be 0.04, 0.16, and 0.31 cm^2^ V^−1^ s^−1^, but significantly increased by factors of 2~4, and was 0.16, 0.28, and 0.95 cm^2^ V^−1^ s^−1^ for the bar-coated PFETs, respectively. Note that the gate leakage currents of the PFETs were maintained below ~10^−9^ A in the bias range. The device parameters are summarized in Table 1. In addition, the aging effect on the device performances of the spin- and bar-coated PFETs was investigated. As shown in Figure S2 of the Supplementary Materials, the PFETs were quite stable up to 30 days: On-current levels (*V_gs_* = −60 V) were almost unchanged and only off-levels (*V_gs_*= ~0 V) were slightly decreased in an order. We considered that these stable operations of the PFETs were attributed to the top gate device geometry, of which the polymeric films were passivated with the top gate dielectric, and also to the subsequent annealing step at a relatively high temperature of 200 °C for 60 min in nitrogen ambience for the films.

**Figure 6:**
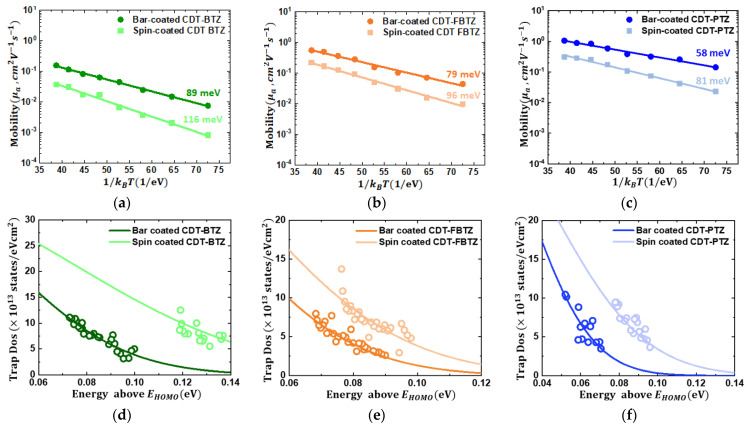
Linear mobility as a function of 1/*T* for the spin- and bar-coated (**a**) CDT-BTZ-, (**b**) CDT-FBTZ-, and (**c**) CDT-PTZ-based PFETs. Extracted charge trap density of states (DOS) of the spin- and bar-coated (**d**) CDT-BTZ-, (**e**) CDT-FBTZ-, and (**f**) CDT-PTZ-based PFETs above the HOMO energy level, *E*_HOMO_. The DOS results were fitted to the Gaussian DOS model.

In an effort to clarify the effect of the microstructural ordering of the CDT-based D-A-type copolymer films on their charge-transport characteristics and electronic structures in more detail, temperature-variable measurements on the CDT Series-based PFETs at temperatures from 160 to 340 K were carried out. As shown in Figure S3 of the Supplementary Materials, the electrical current of the PFETs increased as the temperature increased, which indicated that charge carriers were transferred via thermally activated hopping [28,29,30]. In accordance with the Arrhenius equation of $\mu =\mu_{0}exp\left(-E_{a}/k_{B}T\right)$ [31], the activation energies (*E_a_*) of the PFETs were extracted and found to be 89, 79, and 58 meV for bar-coated CDT-BTZ, CDT-FBTZ, and CDT-PTZ PFETs, whereas those were 116, 96, and 81 meV for spin-coated PFETs, as in Figure 6a–c. The activation energy indicates the required energy for hopping transfer between localized states; thus, the reduced hopping energies for the bar-coated CDT-based PFETs implied that the electronic states of the bar-coated PFETs were more delocalized than those of the spin-coated PFETs. Moreover, in order to ensure the enhanced electronic states of the bar-coated PFETs, the density of states (DOS) of the films was extracted, using the relation as below [32,33,34]:

(2) $$g\left(E\right)=\frac{N}{\sigma \sqrt{2\pi }}exp\left(-\frac{E^{2}}{2\sigma^{2}}\right),$$

where N is the total concentration of charge traps and σ is the width of the trap distributions. Figure 6d–f shows the estimated density of states (DOS) of the spin- and bar-coated PFETs, respectively. It is clearly seen that the trap states of the bar-coated PFETs are more narrowly and shallowly (more delocalized) distributed than those of the spin-coated PFETs. The width of the trap distributions (σ) were estimated to be 153, 94, and 93 meV for the spin-coated CDT-BTZ, CDT-FBTZ, and CDT-PTZ PFETs, whereas it decreased to be 95, 79, and 65 meV for the bar-coated PFETs, respectively. In addition, the total concentrations of the charge traps (N) were estimated to be 6.6, 4.3, and 4.0 × 10^13^ cm^−2^ for the spin-coated CDT-BTZ, CDT-FBTZ, and CDT-PTZ PFETs, whereas they significantly decreased to be 4.2, 3.1, and 3.0 × 10^13^ cm^−2^ for the bar-coated PFETs. As mentioned, these remarkable enhancements in the electronic structures of the bar-coated, CDT-based polymeric films were attributed to the microstructural ordering through the bar-coating-driven gradual drying [21,35]. Semiconducting polymers consist of a long molecular chain that is twisted and a tangled wire, of which disordered phases inevitably exist in the film. The disordered phases would be the source of trap sites that impede charge transport in the film. Thus, it is highly desirable to convert the disordered phases into the ordered phase for high-performance PFETs. As identified with atomic force microscopy, UV/Vis absorption spectra, and charge-transport analyses, the molecular ordering was considerably improved by the bar-coating; thus, the charge-transport characteristics of the bar-coated CDT-based PFETs were significantly enhanced.

## 4. Conclusions

In this study, the structural characteristics and charge-transport behaviors of the spin- and bar-coated CDT-based conjugated copolymers, CDT-BTZ, CDT-FBTZ, and CDT-PTZ, were investigated. Compared to the spin-coated films, the bar-coated polymeric displayed remarkable improvements in molecular structures: Aligned macroscopic structures and nano-fibril microstructures with large grains were observed by optical and atomic microscopic analyses. In addition, the enhanced molecular ordering of the bar-coated polymeric films was identified by UV/Vis absorption spectra analyses, of which additional peaks and red shifts definitely were exhibited in their intramolecular peaks. These improved structural characteristics were regarded to be attributed to the gradual drying from the edge to the center of the bar coating, and led to the high-performance PFETs. As expected, the electrical characteristics of the bar-coated PFETs were remarkably enhanced: The hole currents (*I_ds_* at *V_gs_* = −60 V) increased by an order of magnitude, and the field-effect mobilities significantly increased by factors of 2~4 compared to those of the spin-coated PFETs. In more detail, the temperature-variable current-voltage analyses of the bar-coated PFETs revealed that the required hopping activation energies significantly decreased and the density of states became narrower and shallower, which obviously were attributed to the molecular ordering. Thus, we concluded that the bar-coating method offers practical benefits for improving the molecular ordering, thereby resulting in the high-performance CDT-based PFETs.

## Figures and Tables

**Table 1:** Summary of device parameters and Gaussian DOS parameters for bar-coated and spin-coated CDT series-based PFETs.

Coating Method		Device Parameter			DOS Parameter		
		*g_m_* (×10^−6^ S)	*m_lin_* (cm^2^ V^−1^ s^−1^)	*V_th_* (V)	*E_A_* (meV)	*N* (cm^−2^)	*σ* (meV)
CDT-BTZ	Bar-coating	3.1	0.16	−25	89	4.2 × 10^13^	95
	Spin-coating	0.6	0.04	−33	116	6.6 × 10^13^	153
CDT-FBTZ	Bar-coating	4.9	0.28	−28	79	3.1 × 10^13^	79
	Spin-coating	3	0.16	−32	96	4.3 × 10^13^	94
CDT-PTZ	Bar-coating	17	0.95	−30	58	3.0 × 10^13^	65
	Spin-coating	6.2	0.31	−33	81	4.0 × 10^13^	93

## Data Availability

The data that support the findings of this study are available from the corresponding authors upon reasonable request.

## Supplementary Materials

The following are available online at https://www.mdpi.com/article/10.3390/polym14010002/s1. Figure S1: The histogram showing the number of devices (PFETs) according to mobility, Figure S2: Comparison of transfer characteristics of PFETs at initial state and after 30 days, Figure S3: Temperature dependence of the transfer curves in the linear regime.
