# Supplementary material for: Enhanced Performance of Cyclopentadithiophene-Based Donor-Acceptor-Type Semiconducting Copolymer Transistors Obtained by a Wire Bar-Coating Method
Source: Polymers (Basel). 2021 Dec 21;14(1):2. doi: 10.3390/polym14010002 (PMC8747689; doi:10.3390/polym14010002)
Supplement: Supplementary file 1 [file polymers-14-00002-s001.zip › polymers-1511599-supplementary.pdf]

Supplementary Materials

Enhanced performance of cyclopentadithiophene-based donor-acceptor-type semiconducting copolymer transistors obtained by a wire bar-coating method

Doyeon Kim,[1‡] *Minho Yoon,*[1‡] *and Jiyoul Lee* [1,2*]

[1] *Department of Smart Green Technology Engineering, Pukyong National University, Busan 48513, Republic of Korea*

[2] *Department of Nanotechnology Engineering, Pukyong National University, Busan 48513, Republic of Korea.*

[‡] D. K and M. Y. contributed equally to this work.

Keywords: cyclopentadithiophene-based conjugated polymer, polymer field-effect transistors, fluorine-fluorine repulsion, charge-transport, Gaussian disorder model

---

* Corresponding author. Electronic mail: jiyoul_lee@pknu.ac.kr (J. Lee)

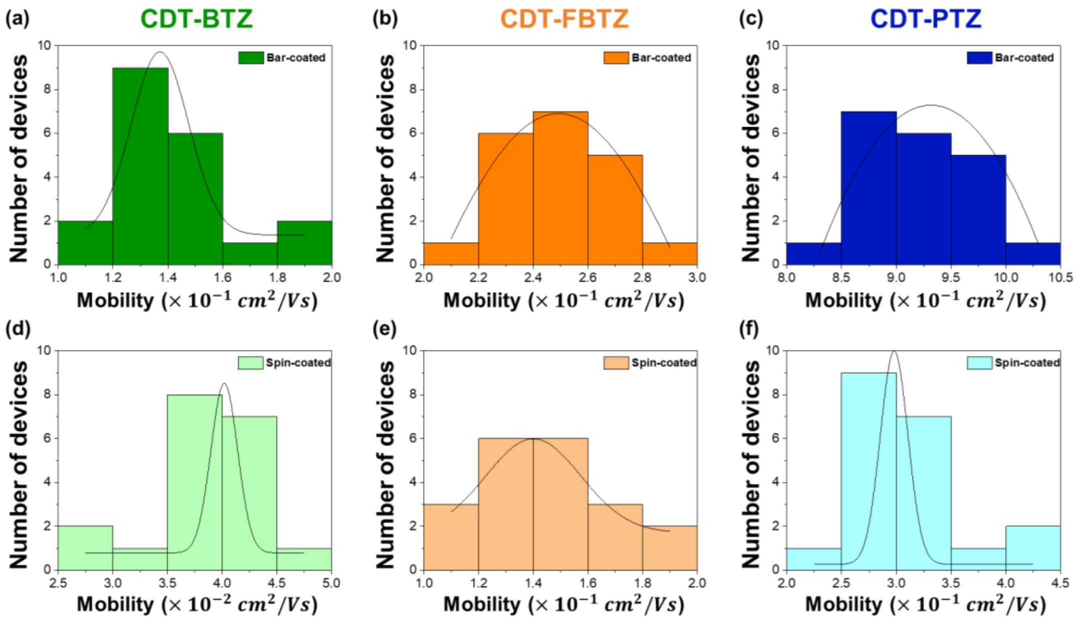

Figure S1. The histogram showing the number of device (PFET) according to mobility for bar-coated (a) CDT-BTZ, (b) CDT-FBTZ, (c) CDT-PTZ based PFETs and spin-coated (d) CDT-BTZ, (e) CDT-FBTZ, (f) CDT-PTZ based PFETs.

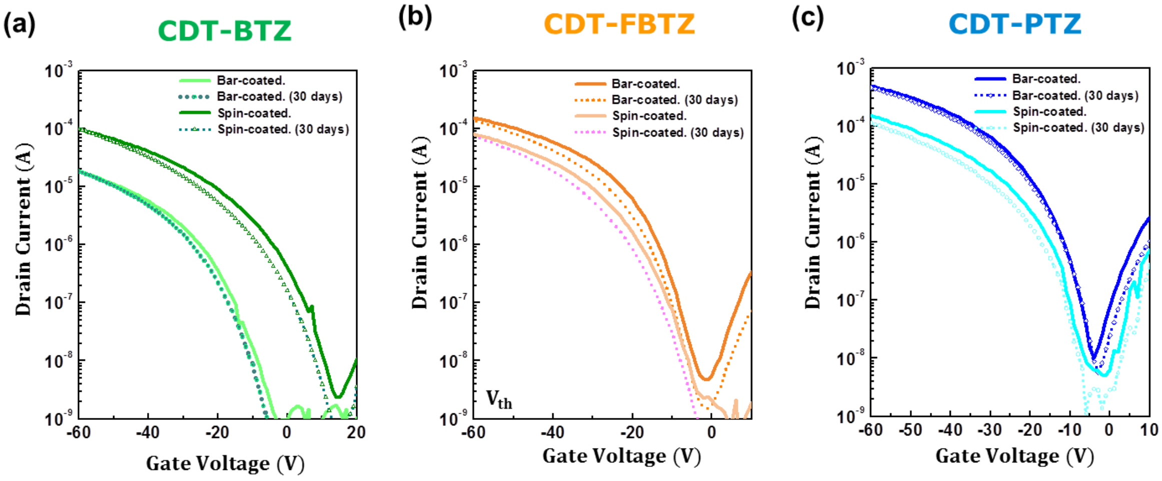

Figure S2. Comparison of transfer characteristics at initial state and after 30 days for the bar-coated and spin coated (a) CDT-BTZ, (b) CDT-FBTZ, (c) CDT-PTZ based PFETs in the linear regime ($V_{DS} = -20$ V).

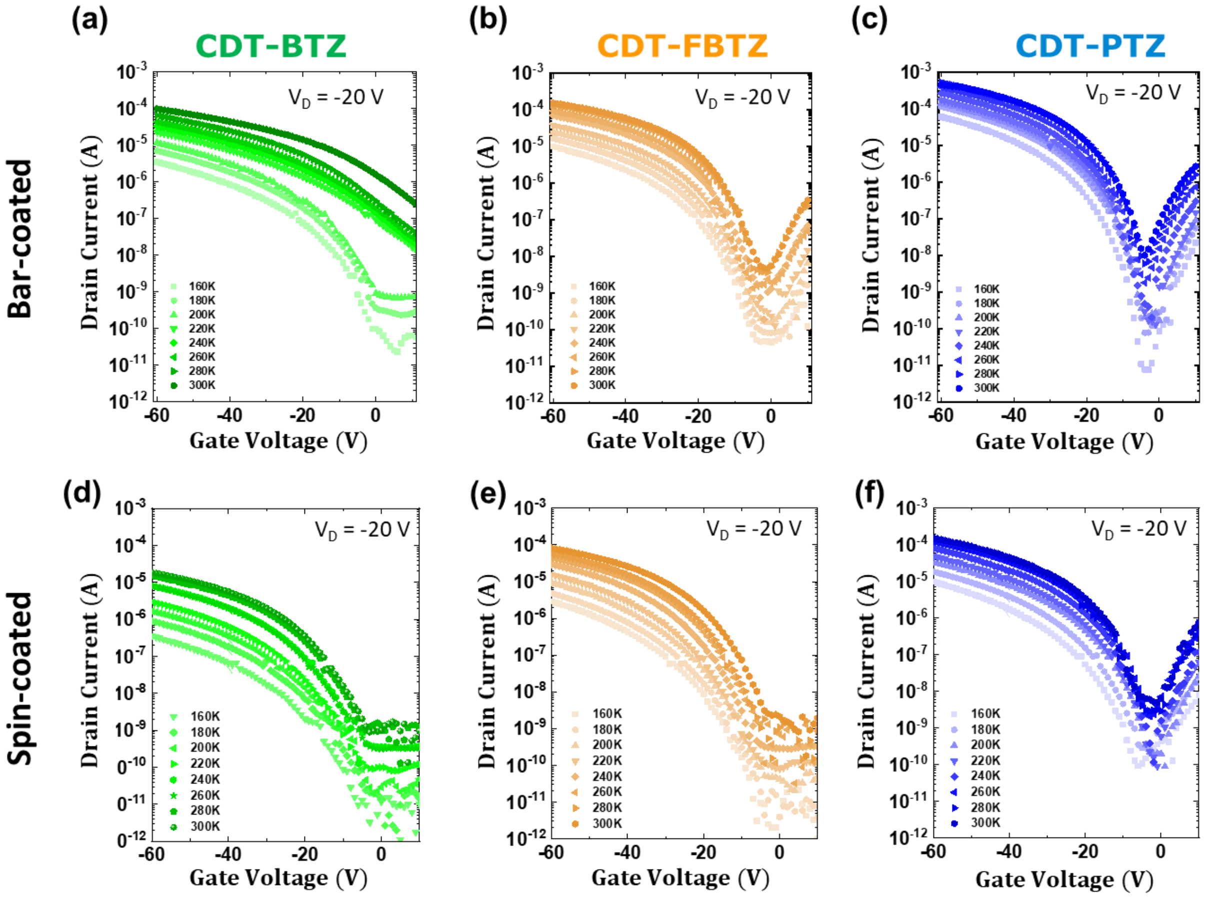

Figure S3. Temperature dependence of the transfer curves in the linear regime ($V_{ds} = -20$ V) for bar-coated (a) CDT-BTZ, (b) CDT-FBTZ, (c) CDT-PTZ based PFETs and Spin-coated (d) CDT-BTZ, (e) CDT-FBTZ, (f) CDT-PTZ based PFETs
